# Supramolecular glucose oxidase-SWNT conjugates formed by ultrasonication: effect of tube length, functionalization and processing time

**DOI:** 10.1186/1477-3155-11-6

**Published:** 2013-02-20

**Authors:** Olukayode Karunwi, Anthony Guiseppi-Elie

**Affiliations:** 1 Center for Bioelectronics, Biosensors and Biochips (C3B), Clemson University Advanced Materials Center, 100 Technology Drive, Anderson, SC 29625, USA; 2 Department of Chemical and Biomolecular Engineering, Clemson University, Clemson, SC 29634, USA; 3 Department of Bioengineering, Clemson University, 29634, Clemson, SC, USA; 4 Department of Electrical and Computer Engineering, Clemson University, 29634, Clemson, SC, USA; 5 ABTECH Scientific, Inc., Biotechnology Research Park, 800 East Leigh Street, 23219, Richmond, VA, USA

**Keywords:** Supra-molecular conjugates, SWNT, Ultrasonic processing, Glucose oxidase, Biosensors, Biofuel cells

## Abstract

**Background:**

Generation-3 (Gen-3) biosensors and advanced enzyme biofuel cells will benefit from direct electron transfer to oxidoreductases facilitated by single-walled carbon nanotubes (SWNTs).

**Methods:**

Supramolecular conjugates of SWNT-glucose oxidase (GOx-SWNT) were produced via ultrasonic processing. Using a Plackett-Burman experimental design to investigate the process of tip ultrasonication (23 kHz), conjugate formation was investigated as a function of ultrasonication times (0, 5, 60 min) and functionalized SWNTs of various tube lengths (SWNT-X-L), (X = −OH or -COOH and L = 3.0 μm, 7.5 μm).

**Results:**

Enzyme activity (K_M_, *k*_cat_, *k*_cat_/K_M_, v_max_ and n (the Hill parameter)) of pGOx (pristine), sGOx (sonicated) and GOx-SWNT-X-L revealed that sonication of any duration increased both K_M_ and *k*_cat_ of GOx but did not change *k*_cat_/K_M_. Functionalized tubes had the most dramatic effect, reducing both K_M_ and *k*_cat_ and reducing *k*_cat_/K_M_. UV–vis spectra over the range of 300 to 550 nm of native enzyme-bound FAD (λ_max_ at 381 and 452 nm) or the blue-shifted solvated FAD of the denatured enzyme (λ_max_ at 377 and 448 nm) revealed that ultrasonication up to 60 minutes had no influence on spectral characteristics of FAD but that the longer SWNTs caused some partial denaturation leading to egress of FAD. Circular dichroism spectral analysis of the 2° structure showed that sonication of any duration caused enrichment in the α-helical content at the sacrifice of the unordered sequences in GOx while the presence of SWNTs, regardless of length and/or functionality, reduced the β-sheet content of pristine GOx. Surface profiling by white light interferometry revealed that ultrasonication produced some aggregation of GOx and that GOx effectively debundled the SWNT.

**Conclusions:**

Supramolecular conjugates formed from shorter, -OH functionalized SWNTs using longer sonication times (60 min) gave the most favored combination for forming bioactive conjugates.

## Introduction

There is pressing need for the design, development and understanding of bio-abio interfaces that permit direct electron transfer of redox enzymes with metallic, carbonaceous or semiconductor electrodes permitting the development of generation-3 (Gen-3) biosensors [1] and advanced biofuel cells [2]. Such biosensors will enable fully implantable continual monitoring of various analytes that serve as markers of a wide variety of physiological conditions and pathologies. Among these are glucose in diabetics [3] and glucose, lactate and succinate in victims of trauma associated hemorrhage [4]. Design and development of Gen-3 biosensors that are reagentless, have long term *in vivo* stability, and require no calibration continues to be a major challenge and opportunity in biomedical diagnostics [5-7]. There is similarly a pressing need for the development of implantable [8,9] biofuel cells that could trickle charge battery powered biomedical devices or to serve as the primary source of power in implantable bioelectronics [10,11]. The biofuel cell [12], which will power the biosensor, can be designed to make use of fuel sources present within the body [13,14]. Both types of biotransduction devices depend upon the design, fabrication and engineering control of biomolecule-to-solid state (bio-abio) interfaces for stable biomolecular recognition [15] and efficient electron transfer [16,17].

Several methods have been proposed over the years to address the foregoing challenge. Among these are generation-1 (Gen-1) biotransducer devices that electrochemically monitor the reactants or products of an enzyme catalyzed reaction, generaton-2 (Gen-2) devices benefit from the use of a free or tethered redox mediators that intercede between charge generation and discharge at an electrode, and Gen-3 biotransducers that allow direct electron transfer across the bio-abio interface [5,18]. Generation-3 biosensors have been achieved by the use of electrical “wiring” using conductive electroactive polymers (CEPs) [19] or the identification of enzymes that facilitate this at nano-structured surfaces [20]. Among the recent novel approaches for establishing an efficient biotransduction mechanism is the use of single walled carbon nanotubes (SWNT)-enzyme conjugates. Guiseppi-Elie et al., in a series of papers [21], were the first to demonstrate direct electron transfer between flavin adenine dinucleotide (FAD) containing glucose oxidase (GOx) [22] and copper containing pseudoazurin [23] at glassy carbon electrodes that was enabled by SWNT-enzyme conjugates formed by adsorption. These exciting results have since generated an avalanche of publications that have ushered in a new vista of study [24]. SWNTs possessing high mechanical properties (tensile strength ~30 GPa, Young Modulus ~1 TPa) and good electrical properties (resistivity of 10^-4^Ωm, maximum current density of 10^13^ A/m^2^) and FAD-containing GOx (β-d-glucose:oxygen 1-oxidoreductase; EC. 1.1.3.4) are suitable model candidates for conjugation and study in the context of Gen-3 biosensors and advanced biofuel cells.

Bioconjugates of GOx-SWNT may be enabled by simple mixing with the supramolecular association being driven by the entropy of mixing and facilitated by the interaction between hydrophobic motifs of the enzyme and the extended pi-structure of the CNTs [25]. However, this is a slow and inefficient process. Guiseppi-Elie et al. have recently reviewed the ever broadening motivations and approaches to forming physical and covalent conjugates between enzymes and SWNTs [24] and have shown that ultrasonic processing [26], the use of cavitation energy, while representing some modest compromise of enzyme activity, may prove a viable route to facilitate rapid and reproducible conjugation of GOx-SWNT suitable for biosensor and biofuel cell applications [27]. Here we expand this work and present detailed investigation of the use of tip ultrasonication (23 kHz) for various sonication times (0, 5 min, 60 min) in the presence of SWNTs of different functionalities (X = −OH or -COOH) and of different tube lengths (L = 3.0 μm, 7.5 μm) (SWNT-X-L), on the activity, stability and structure of GOx component of the conjugate. The activity of the enzyme was monitored by HRP-coupled colorimetric bioassay, UV–vis spectroscopy has been used to monitor the association of the FAD with its apoenzyme, circular dichroism (CD) and white light interferometric imaging have been used to monitor changes in the secondary structure of the enzyme within the GOx-SWNT conjugate.

## Results and discussion

**Figure 1:**
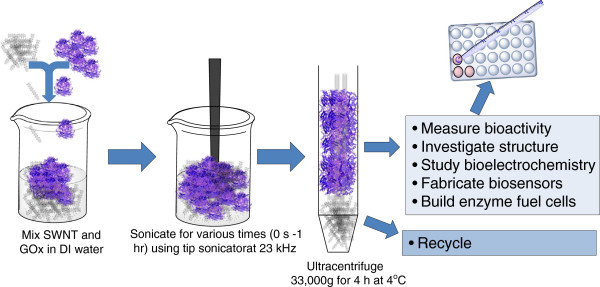
Supra-molecular complex formation via ultrasonic processing and ultracentrifugation.

Ultrasonication at 23 kHz has been known to cause an increase in temperature (up to ~35°C) within 5 minutes and prolonged sonication can increase the temperature of aqueous solutions up to more than 60°C [28]. To eliminate the possible confounding effects of thermal denaturation, all samples were ultrasonicated at ice bath temperatures. Figure 1 summarizes the several steps in GOx-SWNT supra-molecular complex formation via ultrasonic processing and ultracentrifugation. Ultrasonication, a process of using cavitation energy to achieve rapid mixing [29], is well known to accelerate enzyme-catalyzed reactions but it also induces enzyme inactivation through a potentiation of structure and activity of native enzymes [30,31]. The challenge in the design of biomaterials for molecular bioelectronics is to engineer such conjugates with a minimum loss of biofunctionality while conferring some strategic technical advantage; in this case, form the GOx-SWNT supra-molecular conjugate with a minimum loss of bioactivity.

The investigation of the influence of tube length, tube functionalization and ultrasonicaton time on enzyme kinetic parameters, FAD-apoenzyme stability, and protein structure, is amenable to a Plackett-Burman design of experiment approach to identify optimum conditions for further investigation [32]. Table 1 shows the parameter space that was defined by low (−) and high (+) input parameter values. Here ultrasonication time (Low = 5 min and High = 60 min), tube functionalization (Low = −OH and High = −COOH) and tube length (Low = 3.0 μm and High = 7.5 μm). The three output variables studied were; i) the K_M_, *k*_cat_ and *k*_cat_/K_M_ of the enzyme conjugate all expressed relative to the pristine GOx as measured under the same conditions, ii) the extent of the blue shift (λ_max_) in the absorbance maximum of the FAD-apoenzyme at 381 and 452 nm, and iii) the sum of α-helix and β-sheet in the CD spectra.

**Table 1:** Design of experiments: Plackett-Burman design of experiments (Minitab16) to identify optimal conditions for the ultrasonic processing of SWNT-GOx supra-molecular conjugates

**Input Parameters**	**High (+)**	**Low (−)**
Ultrasonication Time	60 minutes	5 minutes
Tube functionality	-OH	-COOH
SWNT length	~7.5 microns (long)	~3 microns (short)
**Output Parameters**		
Enzyme activity	K_M_, *k*_cat_ and *k*_cat_/K_M_	
Enzyme stability	FAD absorption, λ_max_	
Enzyme structure	Sum of α-helix and β-sheet	

### Assays of enzymatic activity

**Figure 2:**
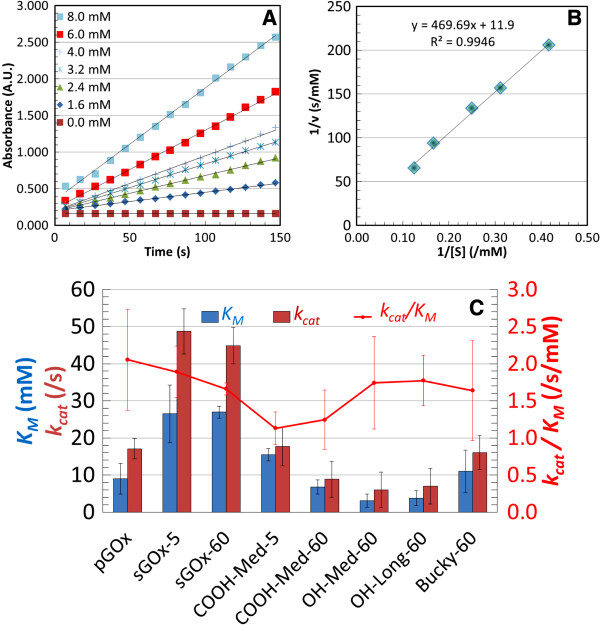
**Enzyme Activity: A) Typical plot of enzyme kinetic data B) Typical Lineweaver-Burk double reciprocal plot from which to extract kinetic parameters. C)** Relationship between K_M_, *k*_cat_, and *k*_cat_ /K_M_ (a reflection of the efficiency of the enzyme) for pristine GOx, sonicated GOx (5 min, 60 min) and selected SWNT-X-L-GOx conjugates. The greatest contribution to change in enzyme efficiency is seen from functionalities of SWNTs.

In order to investigate the effect of ultrasonication on enzyme activity, enzyme activity assays of pristine GOx, sonicated GOx and the various GOx-SWNT-X-L conjugates were determined using the colorimetric GOx-HRP coupled enzymatic assay method. Because enzyme function and fidelity of the active site are strongly connected to the overall structure of the enzyme, these experiments were critical in rationalizing the retained biological activity following ultrasonication as well as after supramolecular conjugate formation by ultrasonication. The enzyme kinetic parameters were determined using varying concentrations of β-d(+) glucose at constant pGOx, sGOx and GOx-SWNT-X-L concentrations and by using both the Lineweaver-Burke and Hill plot methods for comparative purposes. Figure 2A shows a typical plot of the enzyme kinetic data, Figure 2B shows a typical the Lineweaver-Burk plot, and Figure 2C shows the aggregate relationship between K_M_, *k*_cat_, *k*_cat_/K_M_ for pristine GOx, sonicated GOx (5 and 60 min) and selected GOx-SWNT-X-L conjugates. Table 2 presents and compares K_M_, *k*_cat_, *k*_cat_ /K_M_ (the specificity constant; a reflection of the efficiency of the enzyme), v_max_ and n (the Hill parameter) for pristine GOx from this work and various literature sources, sonicated GOx and the various sGOx-SWNT-X-L conjugates.

**Table 2:** **Enzyme activity: presents and compares v**_**max**_**, K**_**M**_**, *k***_**cat**_**, *k***_**cat**_**/K**_**M **_**(a reflection of the efficiency of the enzyme) and *n *(the hill parameter) for pristine GOx, sonicated GOx and the various GOx-SWNT-X-L conjugates**

	**v**_**max **_**(mM/s)**	**K**_**M **_**(mM)**	***k***_**cat **_**(/s)**	***k***_**cat**_**/K**_**M **_**(/mM·s)**	**n**
Pristine GOx	0.00344 ± 0.0008	9.01 ± 4.1	17.1 ± 2.7	2.06 ± 0.68	1.86 ± 0.08
Sonicated GOx-5 min	0.00916 ± 0.0010	26.5 ± 7.8	48.7 ± 6.1	1.89 ± 0.35	1.37 ± 0.10
Sonicated GOx-60 min	0.00888 ± 0.0009	27.0 ± 1.6	44.8 ± 4.9	1.66 ± 0.082	1.46 ± 0.10
GOx-COOH-medium-5 min	0.00351 ± 0.0009	15.5 ± 1.7	17.7 ± 5.3	1.13 ± 0.22	1.14 ± 0.13
GOx-COOH-medium-60 min	0.00190 ± 0.0008	6.82 ± 1.9	8.86 ± 4.8	1.25 ± 0.40	1.82 ± 0.17
GOx-OH-medium-60 min	0.00115 ± 0.0008	3.11 ± 1.8	5.97 ± 4.8	1.74 ± 0.62	1.57 ± 0.05
GOx-OH-Long-60 min	0.00140 ± 0.0008	3.76 ± 2.0	7.01 ± 4.7	1.77 ± 0.34	2.93 ± 0.06
GOx-Bucky-60 min	0.00322 ± 0.0009	11.0 ± 5.7	16.1 ± 4.6	1.64 ± 0.67	1.40 ± 0.19

(Error bars ± 95% confidence interval).

Sonication of pristine GOx, whether for 5 or 60 minutes, produced a significant increase in both K_M_ and *k*_cat_. However, the specificity constant (*k*_cat_/K_M_) was not significantly altered. That is, the effect of sonication was to change the specific rate and the affinity in the same direction and magnitude, and this result was produced whether a short time (5 min) or a long time (60 min) was used. In all cases, the presence of SWNTs was to attenuate the magnitude of change induced by these sonication effects.

Looking at the trend in the specificity constant (*k*_cat_/K_M_), sonication times did not have as much of an influence as did the length and functionality of the SWNTs. The –OH functionalized SWNT (SWNT-OH), although of lower K_M_ and *k*_cat_ values, retained higher enzyme efficiency compared to SWNT-COOH. The length of the tubes did not have as much of an effect on enzyme efficiency. Sonication times likewise did not affect overall efficiency of the enzyme. The extent of sonication, whether short (5 min) or long (60 min) appears inconsequential to enzyme activity under any circumstance. One reason the SWNT-OH conjugates had greater enzyme efficiency than the SWNT-COOH could be due to the carboxyl groups strongly interacting with the amine groups found on the surface of the enzyme thereby slightly altering access to the active site of the enzyme. The sonication times had little effect on the overall enzyme efficiency and this is thought to be a result of the energy damping effects conferred by the high aspect ratio SWNTs. That is, the tubes effectively absorbed and dissipated the cavitation energy that would otherwise induce denaturation/aggregation of the GOx.

Globular proteins such as GOx present an abundance of their polar amino acid side groups on the surface of the enzyme. Functionalized tubes rich in –OH and -COOH groups will strongly hydrogen bond and electrostatically interact with these groups creating conjugates that are topological. There is a strong possibly that these will affect the active site of the enzyme while not gaining proximal access to the deeply buried cofactor. Non-functionalized SWNTs however will more likely interact with the hydrophobic motifs of the enzyme that are deeply buried and proximal to the cofactor.

### UV/Vis spectroscopy of FAD-apoenzyme association

**Figure 3:**
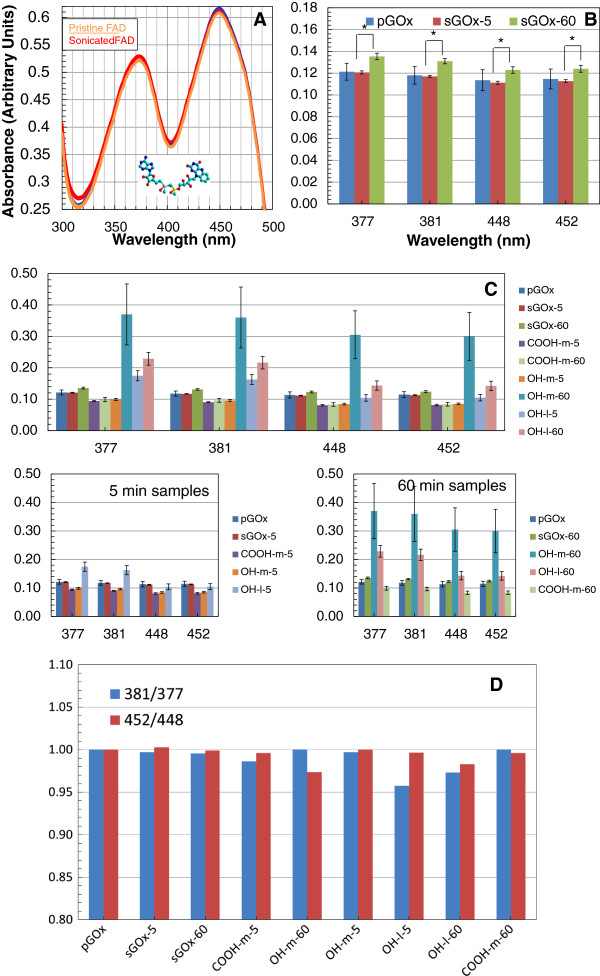
**Enzyme Stability: A) Triplicate measures of the UV/Vis spectra of pristine and sonicated (5 min) Flavin Adenine Dinucleotide (FAD) in DI water (0.1 mg/mL). B)** Changes in the FAD signature in pristine GOx: Bound at 381 and 452 nm; Denatured at 377 and 448 nm. * p < 0.05. **C)** Changes in the FAD signature in GOx-SWNT-X-L: Bound at 381 and 452 nm; Denatured at 377 and 448 nm. **D)** Ratio of 381 nm to 377 nm and 452 nm to 448 nm in the FAD signature in GOx-SWNT-X-L: Ratio in pristine GOx normalized to 1.

In order to determine the effect of ultrasonication on sonicated GOx, including the possible partial aggregation, partial denaturation, or ultrasonically induced self-chemical modification involving reactions of its UV-absorbing amino acid residues, UV–vis absorption spectrophotometric analyses were performed. The UV–vis spectra over the range of 300 to 550 nm was specifically investigated to obtain information pertinent to the binding state of the FAD bound to the native enzyme (absorption maxima at 381 and 452 nm) or the spectrum of the solvent denatured enzyme (FAD absorption maxima at 377 and 448 nm). A blue shift is expected [33] on going from enzyme-bound FAD to free solvated FAD and apoenzyme in solution. This is important to ensure supramolecular conjugates were not denatured by losing FAD. The UV–vis absorption spectra of pristine FAD and sonicated FAD were measured in triplicate. Figure 3A shows that there is no change in the FAD signature upon the application of ultrasonication energy typically used in creating these supramolecular conjugates. This confirmed that ultrasonication, up to 60 min had no influence on spectral characteristics of FAD in solution. The UV–vis absorption spectra of pristine GOx, ultrasonicated GOx (5 min), and ultrasonicated GOx (60 min) in the presence of SWNTs of different functionality and lengths were similarly measured in triplicate. Spectral peaks were deconvoluted and the absorbances at λ_max_ 381 nm and 452 nm (FAD bound in GOx) and λ_max_ 377 nm and 448 nm (partially denatured GOx), expressed as ratios 381 nm / 377 nm and 452 nm / 448 nm, as a measure of the extent of denaturation by ultrasonication times, SWNT lengths and SWNT functionalities. Figure 3B shows that there was some statistical difference (p < 0.05) between the low time (5 min) of sonicated GOx and high time (60 min) of sonicated GOx but no statistical difference between each sonicated GOx system compared to pristine GOx.

Similar to the effect of sonication time on enzyme kinetic parameters, sonication appears to induce some subtle changes in the distribution among FAD-associated and FAD de-associated enzyme; the extent of sonication however, whether 5 min or 60 min, appears inconsequential to this change. This suggests that some number of GOx molecules may be vulnerable to the influence of the disruptive force brought on by the cavitation energy of sonication.

A greater effect on FAD egress (enzyme denaturation) was seen from the influence of tube lengths rather than from the tube functionalities of the SWNT and/or the sonication times. This effect is better shown in the plot of the ratios of bound to free FAD (Figure 3C and 3D) where the greatest change is seen among the varying lengths of the SWNTs rather than functionality or ultrasonication time.

### Circular dichroism spectroscopy for the structure of GOX-SWNT conjugates

**Figure 4:**
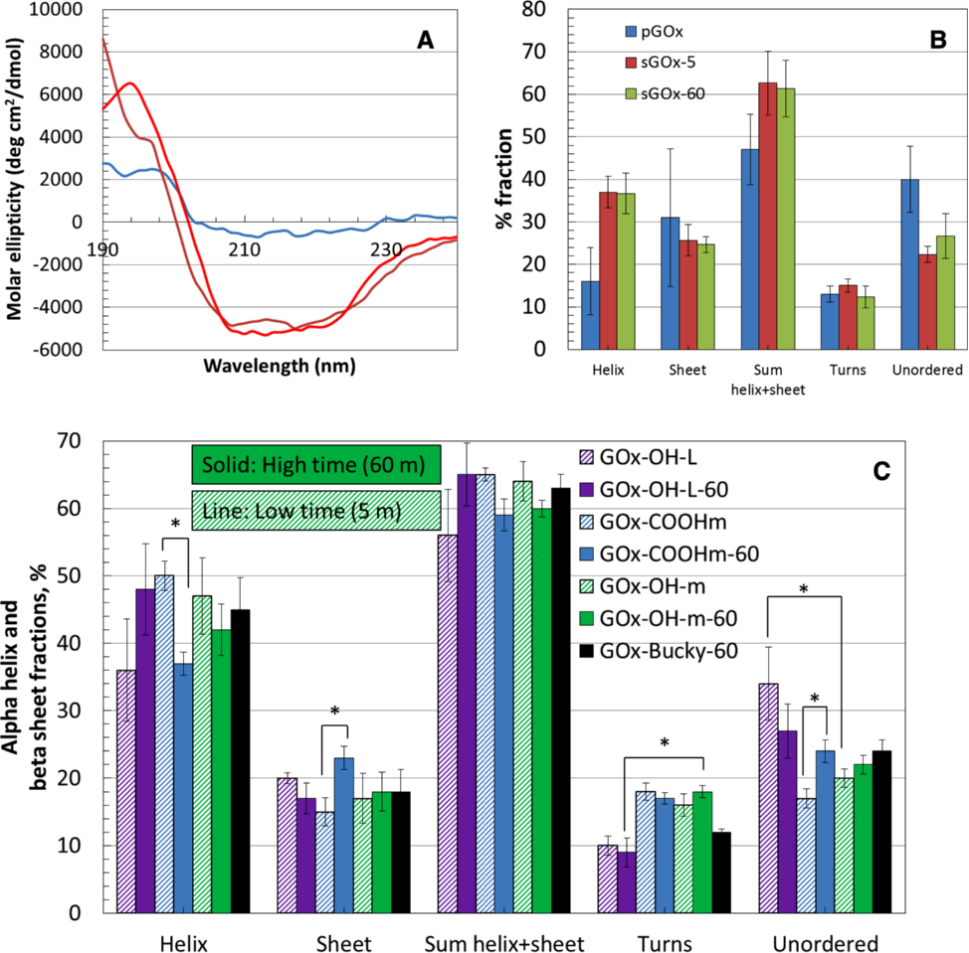
**Enzyme Structure: A) Far-UV CD spectra of pristine GOx. B)** Changes in the secondary structure of GOx following ultrasonication for 5 and 60 min compared to pristine GOX (0 min). * p < 0.05. **C)** Changes in the secondary structure of GOx-SWNT-X-L. Among the three parameters of interest, length of SWNT had the greatest effect on structure compared to functionality and sonication time. * p < 0.05.

Circular dichroism was used to study the changes to 2° structure that accompanied ultrasonication of GOx, both in the absence and presence of SWNT variants. To understand the conformational changes of the pristine (p) and ultrasonicated (s) samples (pGOx, sGOx, GOx-SWNT-X-L) plots of molar ellipticity *vs.* wavelength were produced (a typical plot is shown in Figure 4A) and from these plots were extracted the fractions of α-helix, β-sheet, turns and random sequences. Table 3 gives the fractions, sum of fractions and the relative change of each structural component relative to pristine GOx. This analysis shows that there was no statistical difference in the “sum of the fractions” when comparing pristine GOx to sGOx (5 min, 60 min) although the α-helix fractions and unordered fractions did show significant difference (p < 0.05) (Figure 4B). Sonication, (whether 5 min or 60 min) causes a significant enrichment in the α-helical content. This occurs at the sacrifice of the unordered sequences with no change in the β-sheet content. Again, we see that time of ultrasonication, 5 min or 60 min, is inconsequential to the structural change of the enzyme.

**Table 3:** Enzyme structure: percentage distribution of secondary structural elements and sum of α-helix and β-sheet fractions in pristine GOx, sonicated GOx (5 min and 60 min) and various GOx-SWNT-X-L conjugates

	**α-Helix**	**β-Sheet**	**Sum**	**Turn**	**Random Coil**
Pristine GOx	16	31	47	13	40
Sonicated GOx – 5 min	37	26	63	15	22
Sonicated GOx – 60 min	37	25	62	12	26
GOx-Bucky – 60 min	45	18	63	12	24
GOx-SWNT-OH-long-5 min	36	20	56	10	34
GOx-SWNT-OH-long-60 min	48	17	65	9	27
GOx-SWNT-OH-medium-5 min	47	17	64	16	20
GOx-SWNT-OH-medium-60 min	42	18	60	18	22
GOx-SWNT-COOH-medium-5 min	50	15	65	18	17
GOx-SWNT-COOH-medium-60 min	37	23	60	16	24

When looking closely at the changes to the structure of GOx in the supramolecular GOx-SWNT-X-L conjugates (Figure 4C), the greatest contributor to a significant change in the secondary structure of the enzyme was tube length. Tube length contributes a significant change (p < 0.05) in the unordered fractions [e.g. OH-M-5 *vs.* OH-L-5 and (Unordered p = 0.024) and OH-M-60 *vs.* OH-L-60 (Unordered p = 0.0006)] of the secondary structure of the enzyme while sonication times (whether 5 min or 60 min) did not show any significant difference. Ultrasonication, as a process, regardless of the presence of SWNTs, causes an increase in the sum of α-helix, β-sheet components at the sacrifice of the unordered sequences. On the other hand, ultrasonication in the presence of SWNTs, regardless of length and functionality, reduced the β-sheet content of pristine GOx suggesting their unambiguous role in disrupting intramolecular hydrogen bonding. SWNTs therefore have a unique interaction with GOx in the presence of ultrasonication that opposes the action of ultrasonication taken alone. In general, ultrasonication times (5 min or 60 min) did not show any significant difference among the various conjugates (similar to the action in the absence of SWNTs) except in the case of COOH-M-5 vs. COOH-M-60 where there was a significant reduction (p = 0.003) in the α-helix content, a significant increase (p = 0.014) in the β-sheet component, and a significant increase (p = 0.011) in the unordered portion with increased ultrasonication time. Thus –COOH functionalized tubes clearly acted uniquely compared to –OH functionalized and Bucky tubes. This shift in ordered fractions can be attributed to the interaction of the carboxyl groups on the SWNTs with the surface amine groups on GOx. Such a difference in mode of action could arise from a release of surface pressure [34] and the induced structural transformations, which may affect the active site of the enzyme.

### Surface profile imaging

**Figure 5:**
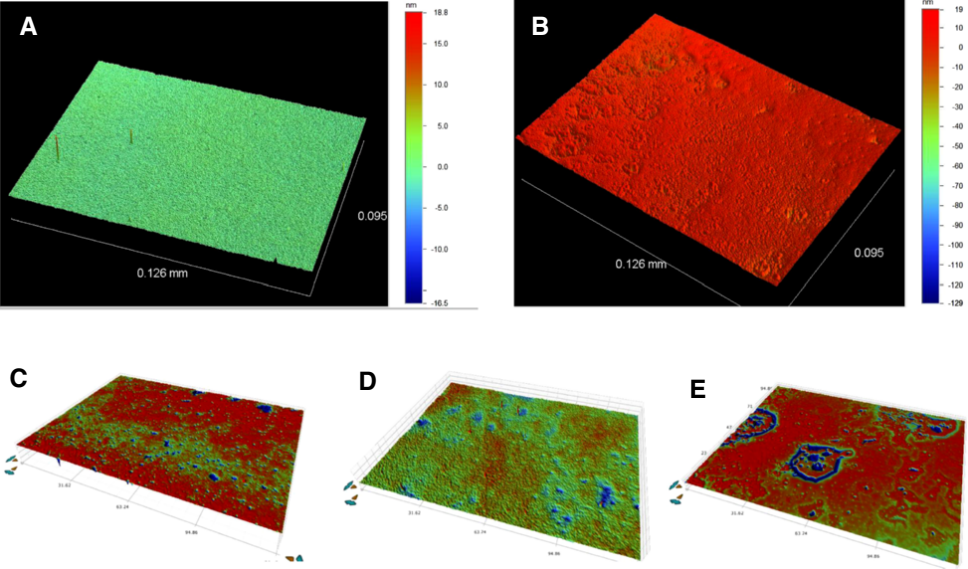
Surface Profile: Surface morphology using non-contact optical profiling of: A) Blank PME 118-Pt, B) Physically adsorbed GOx (20 μL of 1 mg/mL) on PME 118-Pt, C) Physically adsorbed GOx-SWNT (20 μL of 1 mg/mL)on PME 118-Pt, D) Physically adsorbed sonicated (60 min) GOx (20 μL of 1 mg/mL) on PME 118-Pt, and E) Physically adsorbed SWNT (20 μL of 1 mg/mL) on PME 118-Pt.

White light interferometry [35-37] was used to produce 3D surface images of adsorbed pGOx, sGOx, and GOx-SWNT-X-L conjugates on platinum electrodes. Figure 5 shows the surface morphology using non-contact optical profiling of: A) blank PME 118-Pt, B) physically adsorbed GOx (20 μL of 1 mg/mL), C) physically adsorbed GOx-SWNT (20 μL of 1 mg/mL), D) physically adsorbed sonicated GOx (60 min; 20 μL of 1 mg/mL) and E) physically adsorbed SWNT (20 μL of 1 mg/mL) on PME 118-Pt. These images clearly show that what was a generally uniform, featureless metallic surface became roughed by the presence of adsorbed GOx aggregates. From these images were extracted average surface roughness (Sa, nm) and the developed surface area ratio (Sdr, unitless) [Sdr = (texture surface area – plan surface area) / plan surface area] and these are tabulated in Table 4. In both cases the data are also referenced to the blank Pt electrode.

**Table 4:** Surface profile: a comparison of the surface profiles of pristine GOx, sonicated (60 min) GOx, SWNT and GOx-SWNT examined as determined by non-contact optical profiling

**Materials**	**Sa (Average Roughness), nm**	**Ratio to blank**	**Sdr (developed surface area ratio)**	**Ratio to blank**
Pt	0.254	1.00	0.114	1.00
Pt|pGOx	1.928	7.59	0.122	1.07
Pt|sGOx-SWNT	9.408	37.04	0.0025	0.02
Pt|sGOx	10.688	42.08	0.0071	0.06
Pt|SWNT	66.222	260.72	0.0431	0.38

The surface roughness may by inference be related to the aggregation state of the protein and SWNT following adsorption and drying on the Pt substrate. The Pt|sGOx and Pt|sGOx-SWNT both show similar surface roughness but produce surface roughness that is ca. 5X that produced by the pristine GOx. This confirms that ultrasonication likely produced aggregation of the GOx, independent of the presence of the SWNT. The structural changes, particularly the increase in α-helix content, are consistent with the formation of GOx aggregates. On the other hand, the Pt|SWNT produced a surface which was 7X that produced by the Pt|sGOx-SWNT. This confirms that the GOx effectively de-aggregates the SWNTs bundles and produces a surface structure that is governed by the protein member of the conjugate pair. This suggests that the SWNTs are effectively individualized, or are at least of bundle sizes less than that of the protein aggregates.

### Optimal processing conditions

The design of experiments approach allows us to rapidly converge upon generally optimized processing conditions for producing GOx-SWNT supramolecular conjugates with minimum loss of enzyme activity for the best retained activity. The experimental design suggested 12 unique experiments that were conducted in triplicate and resulted in 36 separate test samples. From an analysis of the experimental data, shorter SWNTs functionalized with –OH groups and provided with longer sonication times (no difference with respect to shorter sonication times) gave the best combination for forming bioactive conjugates. There are other issues of course. For example, are these short, –OH functionalized SWNTs conductive? Can the bioactive conjugate support direct electron transfer? Can these bioactive conjugates be immobilized onto solid or porous electrodes to promote direct electron transfer appropriate for Gen-3 biosensors and advanced biofuel cells?

## Conclusions

These investigations reveal that tube length of SWNTs has the greatest overall effect compared to sonication times and functionalities on FAD retention and enzyme structure while functionality of the SWNT has a greater effect on the kinetic efficiency of the enzyme. A high level of enzyme activity was conserved for all conjugates. Shorter SWNTs supported conjugate formation with no loss of FAD and conserved enzymatic structure while the longer SWNTs caused some partial denaturation leading to the egress of FAD. Ultrasonication, regardless of time used short or long, promotes GOx aggregation as evidenced by the increase in α-helix content and the surface roughness data. Ultrasonication, as a processing technique, has an almost instantaneous effect on GOx structure and activity that appears to be the associated with aggregate formation. SWNT stabilizes the GOx from ultrasonic denaturation by absorbing and dissipating portions of ultrasonic energy put into creating conjugates. Future studies will characterize the electron transfer kinetics as well as perform amperommetry measurements to determine efficacy in biosensors and biofuel cells. In addition, long term viability studies will be run to ensure the implantable biosensors have a relatively long shelf life.

## Experimental methods

### Materials and reagents

SWNTs (purity, 95 wt.%) were purchased from Bucky USA (Houston, TX, USA) and were used as received. These tubes were un-functionalized and un-sorted by length and were referred to as Bucky tubes or simply Bucky in this manuscript. Functionalized SWNTs possessing –OH and –COOH groups with two different tube lengths (3.0 μm, 7.5 μm) were purchased from NanoLab, Inc. (Waltham, MA, USA) and used as received. In summary, SWNTs-OH and SWNTs-COOH of different lengths (~3.0 μm and ~7.5 μm) were first produced using chemical vapor deposition (CVD) of a carbon-carrying feedstock [methane (CH_3_)] delivered at a controlled rate over a catalyst bed of iron nanoparticles deposited on a fumed silica support at 700°C. SWNTs were subsequently purified in HF/HCl and the resulting product rinsed in deionized water until pH neutral, then drained and annealed. SWNTs were produced with high purity and with little or no amorphous carbon through careful control of the catalyst size, process time, temperature, and pressure [38]. SWCNTs were produced at 1–1.5 nm in diameter with ~7.5 μm of length and subsequently ball milled to produce the shorter version (~ 3.0 μm) so that the chemical composition of the two different lengths would be the same. Energy-dispersive X-ray spectroscopic analysis (SEM-EDX) provided by the manufacturer confirmed that the SWCNTs contained 95.93 wt% carbon and 4.07 wt% of other elements (including Na, Al, Si, S, and Fe). End and sidewall -COOH functionalization (1.0-7.0 atom%) was achieved by the use of 1:3 HNO_3_:H_2_SO_4_, 8 M acid under refluxing conditions (~80°C, 4 hours). For end and sidewall -OH functionalization (1.0–7.0 atom%), the carboxylated nanotubes are returned to the reflux apparatus but this time in a KOH solution.

Glucose oxidase (EC 1.1.3.4 from *Aspergillus niger*, G7141-250KU, type X-S, 146,000 units/g solid; lyophilized powder containing approximately 75% protein) was purchased from Sigma Aldrich (St. Louis, MO, USA) and used as received. For the high-speed centrifugation of the GOx-SWNT suspension after ultrasonication, ultracentrifuge tubes (OakRidge Bottle, polycarbonate 16 × 83 mm and polypropylene sealing caps) were purchased from Fisher Scientific (Pittsburg, PA, USA). Horseradish peroxidase (HRP) (EC 1.11.1.7, P-8250-50KU, type II, 60 purpurogallin units/mg solid), sodium acetate buffer 2,2'-azino-bis(3-ethylbenzothiazoline-6-sulphonic acid) (ABTS) and β-d(+)-glucose substrate solution were purchased from Sigma (St. Louis, MO, USA). The 96-well plates for the enzymatic bioassays were purchased from Falcon (BD Biosciences, Franklin Lakes, NJ USA) and DI water was generated by a Milli-Q® Ultrapure Water Purification System.

### Preparation of the GOx-SWNT conjugate dispersions

Aqueous suspensions of appropriate weights of functionalized SWNTs of various lengths (SWNT-X-L where X = −OH or –COOH groups and L = 3.0 μm 7.5 μm) (5 mg) and GOx (5 mg) were prepared in 5.0 mL of DI water (1 mg/ml each component). Suspensions were prepared by ultrasonication at 4°C within a jacketed water bath using a Soniprep 150 (MSE, UK) equipped with an MSE exponential probe (tip diameter 3 mm, transformation ratio 7:1) ultratip sonicator (frequency 23 kHz) at two different time intervals, 5 min and 60 min (0 min (control)). The GOX-SWNT conjugates were collected from the supernatant following high-speed centrifugation (33,000 × g for 4 h at 4°C) using a high-speed Sorval Evolution RC Centrifuge (Thermo Scientific, IL, USA) equipped with a SS-34 rotor [27]. Following centrifugation, all samples were collected and stored in the refrigerator at 4°C. Figure 1 illustrates and summarizes this procedure. TEMs images of SWCNT-GOx conjugates prepared by this method have been previously published [27].

### Enzyme assays

**Figure 6:**
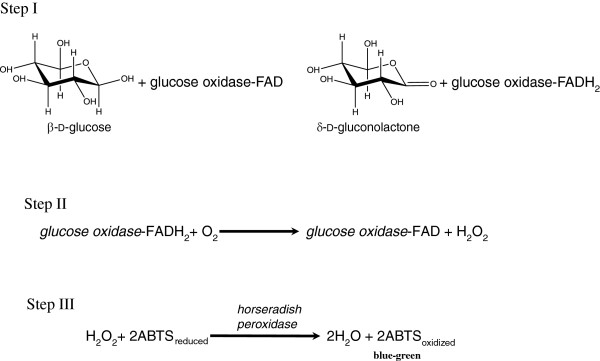
Assay of glucose oxidase activity via an HRP-linked colorimetric response using ABTS.

All samples containing GOx were subjected to the standard HRP-coupled enzymatic assay [39] protocol recommended by Sigma Aldrich. This assay is a coupled enzyme assay wherein the enzyme catalyzed reaction is linked to the decomposition of hydrogen peroxide with horseradish peroxidase (HRP) and the oxidation of the chromogenic reagent, 2,2'-azino-bis(3-ethylbenzothiazoline-6-sulphonic acid) (ABTS). ABTS was used in place of *o*-dianisidine for more stable results. Figure 6 shows the three step process for generating the colorimetric glucose dose response.

The following reagents were prepared and used for the enzymatic assay experiments: A, 48 mM sodium acetate buffer, pH 5.1 (adjusted with 1 M HCl) at 37°C which contained 0.1 mg/ml of sodium azide; B, 0.16 mM ABTS in reagent A; C, varying concentrations of mutarotated β-d (+) glucose substrate in DI water; D, reaction cocktail consisting of reagent B and C (24:5 v/v), equilibrated to 37°C and adjusted to pH 5.1 with 1 M HCl or 1 M NaOH; E, HRP solution containing 60 purpurogallin units/ml in DI water; F sample of interest (pristine GOx, GOx-SWNT conjugate, etc.). The reactions were carried out by mixing 91.9 μl of freshly prepared D and 68.1 μl of E (contains 6 units HRP) in a 96-well plate. The mixture was equilibrated at 37°C in the plate reader and the absorbance at 405 nm monitored for about 10 minutes until it was constant. Finally, 40 μl of F (contains 0.004 mg solid/ml GOx) was added and the increase in absorbance at 405 nm was monitored every 10 s for about 5 min. Using the maximum/initial linear rate yielded the change in absorbance per minute which was converted to units of enzyme activity (μmol/min) using an extinction coefficient of 36.8 mM^-1^ cm^-1^ at 405 nm [40]. One enzyme unit is defined as the amount of enzyme required to oxidize 1 μmol of β-d glucose per minute at 37°C. The initial rate data was used to determine the enzyme kinetic parameters (v_*max*_*, K*_*M*_*, n, k*_*cat*_*,* and *k*_*cat*_*/K*_*M*_) through nonlinear curve fitting of the Hill function (Equation 1), and where appropriate, the Lineweaver-Burk plot in conjunction with the Michaelis-Menten equation.

(1) $$v=v_{\text{max}}\left(\frac{c_{s}{}^{n}}{K_{M}{}^{n}+C_{s}{}^{n}}\right)$$

Statistical analysis of triplicate data via *t*-test statistic was used to establish a *p*-value.

### UV/Vis spectroscopy

UV–vis spectroscopy was performed using a Synergy Mx Monochromator-based Multi-mode Microplate Reader running Gen5 software. For this, 200 μl of each of the ultrasonicated aqueous GOx-SWNT conjugate solution and control samples (pristine GOx, sonicated GOx without SWNT) was placed in a 96-well plate and the UV/Vis absorption spectra recorded over the wavelength range 230 – 900 nm. The range 300 – 500 nm was specifically isolated and analyzed for its relevance to FAD-apoenzyme association [41,42]. Since many buffers and common buffer additives have a strong absorbance in the far UV region, the aqueous GOx-SWNT and control sample solutions were prepared buffer free.

### Circular dichroism spectroscopy

The Circular Dichroism (CD) [43] measurements were performed at 25°C in a 1.0 cm quartz cuvette (Stama Cells, Atascadero, CA, USA) over the wavelength range 190–300 nm on a Jasco J-810 spectropolarimeter (Jasco, Easton, MD, USA) fitted with a xenon lamp. Each scan was the average of six accumulations using a scan rate of 2 nm/min and 0.1 nm resolution. The CD spectra for 0.005 mg/mL concentration of GOx solution and those of the various GOx-SWNT-X-L conjugates were obtained in nanopure water. These spectra were then deconvoluted using the CDPro software package and the secondary structural components for the native protein and the nanotube-protein conjugates were determined using the CONTINLL-4, CONTINLL-7 [44], CDSSTR-4, CDSSTR-7 [45] computer program. Data cut was applied to the CD spectra (data pitch: 0.1 nm) as well as smoothing to the final curves. A mean residue weight of 110 Da was used for GOx while calculating the molar ellipticity [*θ*]_MRW_ (in deg cm^2^/dmol).

### Surface profile imaging

An automated Contour GT-K1 Optical Profiler (Bruker Nano Surfaces Division, USA) was used to provide high resolution 3D surface images and obtain surface roughness using white light interferometric technology [35]. Composites were prepared on 1.0 cm × 2.0 cm × 0.05 cm platinum planar metal electrodes (PME 118-Pt; ABTECH Scientific Inc., Richmond, VA, USA) and imaged under dry conditions. The samples imaged include: i) Blank PME 118-Pt, ii) Physically adsorbed GOx (20 μL of 1 mg/mL) on PME 118-Pt, iii) Physically adsorbed GOx-SWNT (20 μL of 1 mg/mL) on PME 118-Pt, iv) Physically adsorbed sonicated (60 min) GOx (20 μL of 1 mg/mL) on PME 118-Pt, and v) Physically adsorbed SWNT (20 μL of 1 mg/mL) on PME 118-Pt.

## Abbreviations

GOx: Glucose oxidase; pGOx: Pristine glucose oxidase; sGOx: Sonicated glucose oxidase; FAD: Flavin adenine dinucleotide; HRP: Horseradish peroxidase; SWNT: Single-walled carbon nanotube; PME: Planar metal electrode; Pt: Platinum;Gen-3: Generation-3; CD: Circular dichroism; kcat: The turnover number of an enzyme; KM: The Michales constant – an inverse measure of the affinity of an enzyme for its substrate

## Competing interests

Both authors declare that they have no competing interests.

## Authors’ contributions

OK carried out the experimental work and drafted the manuscript. AG-E conceived of the study, directed its design and coordination and finalized the manuscript. All authors read and approved the final manuscript.
